# COVID-19 vaccine immunogenicity in Mongolian adults with and without chronic hepatitis

**DOI:** 10.1186/s12879-026-13095-y

**Published:** 2026-03-24

**Authors:** Viviane Callier, Ganbolor Jargalsaikhan, Munguntsetseg Batkhuu, Renee Ridzon, Sally Hunsberger, Irini Sereti, Kathryn Shaw-Saliba, Shera Weyers, Alyson Francis, Naranjaral Dashdorj

**Affiliations:** 1 Clinical Monitoring Research Program Directorate, Frederick National Laboratory, Frederick, MD USA; 2 Onom Foundation, Ulaanbaatar, Mongolia; 3 Liver Center, Ulaanbaatar, Mongolia; 4 Henry M. Jackson Foundation for the Advancement of Military Medicine, Inc., Bethesda, MD USA; 5 National Institute of Allergy and Infectious Diseases, National Institutes of Health, Bethesda, MD USA; 6 Infinity Bio, Inc., Baltimore, MD USA; 7 Systex, Inc., Rockville, MD USA

**Keywords:** hepatitis, COVID-19, Vaccine, Immunogenicity, Mongolia

## Abstract

**Background:**

Mongolia has a high prevalence of liver disease from chronic viral hepatitis infections, which can compromise the immune response. It is unclear whether people with chronic liver disease produce a robust antibody response to the COVID-19 vaccines. We investigated whether the antibody response to the COVID-19 booster vaccination differed between those with and without chronic viral hepatitis from multiple viral types.

**Methods:**

Participants from Mongolia were recruited to the International Study on COVID-19 Vaccine to Assess Immunogenicity, Reactogenicity and Efficacy (InVITE). Demographic information was recorded and participants’ SARS-CoV2 anti-Spike antibody levels were measured at visit 1 (prior to administration of booster vaccine at baseline) and visit 2 (approximately 2 +/-1 months after booster vaccination). We used linear regression to determine if the anti-Spike antibody responses differed between participants with and without different types of chronic viral hepatitis infections.

**Results:**

Overall, there was no significant difference in the anti-Spike antibody response between participants with and without chronic viral hepatitis, a result suggesting that COVID-19 vaccine booster immunogenicity is not affected by chronic hepatitis. Participants with chronic HCV infection produced a robust antibody response, but based on our linear regression analysis, this response seemed to wane faster than in participants without chronic viral hepatitis.

**Conclusions:**

Our finding that there was no significant difference in the anti-Spike antibody response between participants with and without chronic viral hepatitis adds to the evidence that the COVID-19 vaccines are immunogenic in people with chronic viral hepatitis, including in those with HBV/HDV coinfection.

**Supplementary Information:**

The online version contains supplementary material available at 10.1186/s12879-026-13095-y.

## Introduction

Vaccination has been an important aspect in the response to the COVID-19 pandemic. The World Health Organization granted emergency use listing to 11 COVID-19 vaccines [23]. Protection conferred by vaccination leads to a decrease in COVID-19 disease severity, hospitalizations and death and antibody response to vaccination is correlated with this protection [14]. The International Study on COVID-19 Vaccine to Assess Immunogenicity, Reactogenicity and Efficacy (InVITE) began in August 2021 (https://clinicaltrials.gov/ct2/show/NCT05096091) and was designed to examine the immunogenicity of different COVID-19 vaccines used in the national immunization programs in seven countries including Democratic Republic of Congo (DRC), Guinea, Indonesia, Liberia, Mali, Mexico and Mongolia [27].

In people who are immunocompromised, the immune response to vaccination may be blunted and vaccine efficacy may be reduced [19, 22, 24]. Chronic liver disease is associated with immune dysfunction [2, 18] and poorer outcomes with COVID-19 [21]. Studies examining immunogenicity of COVID-19 vaccines have noted suboptimal responses in persons with chronic liver disease and liver transplantation [13, 30], however, these studies have not specifically looked at the impact of chronic viral hepatitis.

Worldwide in 2022, 304 million people were living with chronic viral hepatitis B and C [32]. 254 million (3.3% of the world’s population) were living with hepatitis B, and about 50 million (0.7% of world’s population) were living with hepatitis C [32]. Asia has a high burden of hepatitis [20] and Mongolia in particular has one of the world’s highest prevalences of chronic viral hepatitis [9, 12]. Based on a nationwide survey conducted in Mongolia in 2017, the estimated prevalence of chronic hepatitis B virus (HBV) and hepatitis C virus (HCV) infection in the adult population was as high as 11.1% and 7.1%, respectively [11]. Approximately 60% of those with chronic hepatitis B virus infection in Mongolia have hepatitis delta virus (HDV) coinfection [6]. In a study that looked at HBV-related deaths in 2019 globally, the highest rate was reported from Mongolia at 29.1/100,000 [9]. This relatively high burden of chronic viral hepatitis, with and without cirrhosis, affords an opportunity to examine the association of chronic viral hepatitis with immune response to COVID-19 vaccination in the cohort of adults from Mongolia enrolled in the InVITE study.

## Methods

Details of the InVITE study design including eligibility criteria and objectives have been previously published [27]. Persons 18 years or older from seven countries who provided informed consent were enrolled within one day of receiving a COVID-19 vaccine either as the primary or as a booster dose.

In addition to standard data collected at enrollment, information about liver disease and chronic hepatitis was collected. The primary endpoint of the study was measurement of SARS-CoV-2 anti-Spike (anti-S) levels. In Mongolia, 1001 participants were enrolled after receiving either a first or second booster COVID-19 vaccine (AstraZeneca, Pfizer, or Sinopharm). Participants were recruited from the general population as well as from patients attending the Liver Center that delivers care to persons with chronic liver disease. The study population includes patients with chronic viral hepatitis as a result of infection with hepatitis C virus, hepatitis B virus and hepatitis B and D virus coinfection. This was a substudy of the InVITE study [27] and only included participants from Mongolia.

**Fig. 1:**
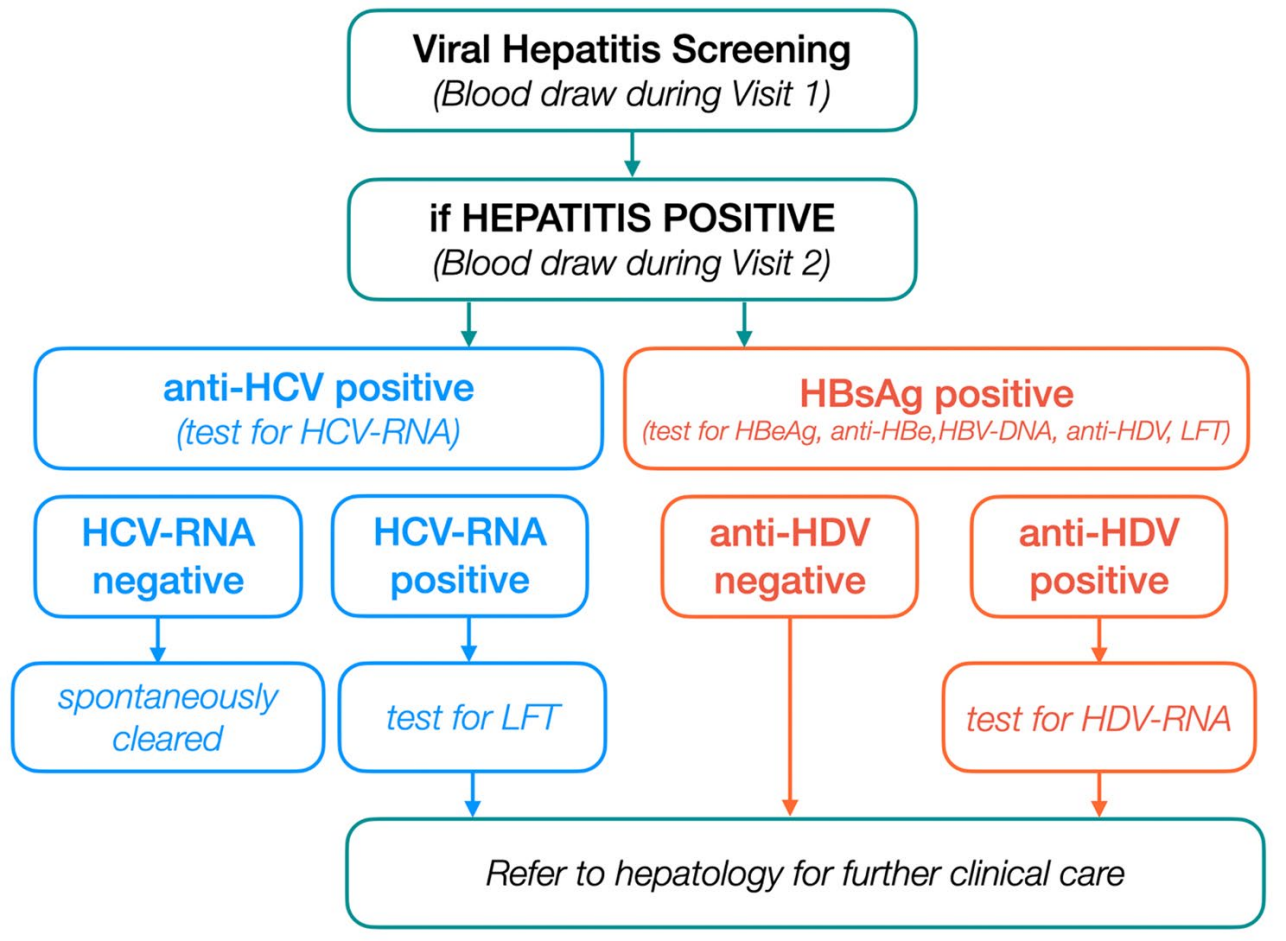
Screening and testing procedures

The presence of chronic viral hepatitis infection was determined with initial screening for Hepatitis B Virus (HBV) and Hepatitis C Virus (HCV) with tests for Hepatitis B surface antigen (HBsAg) (HISCL-5000, Sysmex, Japan), and anti-HCV antibody (HISCL-5000, Sysmex, Japan) (CDC [5,8, 29, 31]). For those with positive anti-HCV, reflex testing for HCV RNA (GenXpert, Cepheid, USA) was performed. For those persons found to have reactive HBsAg, reflex testing for both HBV-DNA (GenXpert, Cepheid, USA) and antibody to Hepatitis D Virus (HDV) (anti-HDV) (Wantai, China) was performed. In-house HDV RNA testing (CFX Opus 96, BioRad, USA) was performed for all persons found to have positive anti-HDV (Fig. 1). Chronic HBV infection was defined as having a positive HBsAg. Chronic HCV infection was defined as having both a positive anti-HCV and HCV RNA, and resolved HCV was defined as having a positive anti-HCV and negative HCV RNA. Chronic HBV/HDV coinfection was defined as having HBsAg with both positive anti-HDV and HDV RNA tests. Chronic viral hepatitis was defined as having chronic HBV, chronic HCV infection or chronic HBV/HDV coinfection. Liver functions were tested in those who had chronic viral hepatitis using automated BX-3010 (Sysmex, Japan). Elevated ALT and AST were defined as higher than two times of upper limit of normal range. Elevated AST was defined as > 70 U/L for women and > 82 U/L for men. Similarly, elevated ALT was defined as > 70 U/L for women and > 82 U/L for men. Elevated bilirubin was defined as > 20 umol/L and decreased albumin was defined as < 35 g/L.

A liver stiffness measurement and controlled-attenuation parameter by transient elastography were performed on all participants using FibroScan^®^Expert 630 (Echosens, France) and results were recorded with steatosis grade (S0-S3) and fibrosis scores (F0/F1-F4). Cirrhosis was defined by the finding of a F4 score on Fibroscan. All persons found to have chronic viral hepatitis or abnormal Fibroscan results were referred for further clinical care.

Measurement of SARS-CoV-2 antibody levels were taken at baseline (Visit 1, at booster vaccination) and participants were asked to return for study Visit 2 approximately 2 +/-1 months after booster vaccination (30–90 days after Visit 1). The intended window for visit 2 was approximately 2 months (60 days) plus or minus 1 month (30 days), however, many visit 2 measurements occurred outside of the preferred study window. Specimens for these measurements were stored at -80 °C within 4 h of collection at the study site in Mongolia and shipped to the central lab (Frederick National Laboratory, Frederick, Maryland, United States of America). SARS-CoV-2 anti-S levels were measured using the Quanterix anti-Spike IgG semi-quantitative antibody assay (Quanterix, Billerica, MA, USA), and total (IgA, IgG, and IgM) SARS-CoV-2 serum anti-nucleocapsid (anti-N) antibody levels were measured with the BioRad Platelia SARS-CoV-2 total Ab assay (BioRad, Hercules, CA, USA) according to the manufacturer’s instructions. Methodological details and specificity of these assays have been described elsewhere [10, 15, 17].

Regression analyses were used to analyze antibody waning over time and estimate the association between anti-S antibody levels (log 10 transformed) and variables indicating liver disease. All regression models included time from visit 1 to visit 2 (we will refer to as time), since many visit 2 measurements occurred outside the preferred study visit window (60 +/- 30 days after Visit 1). In all regression models, we initially included an interaction between time and the liver disease variables of interest. If the interaction was not significant (*p* > 0.05), the interaction term was dropped from the model. We performed simple regression models including only time and a liver disease variable and a model that adjusts for more baseline covariates including baseline anti-N antibody positivity, sex, body mass index (BMI), age, vaccine type, and time from last pre-study COVID-19 vaccine.

## Results

**Fig. 2:**
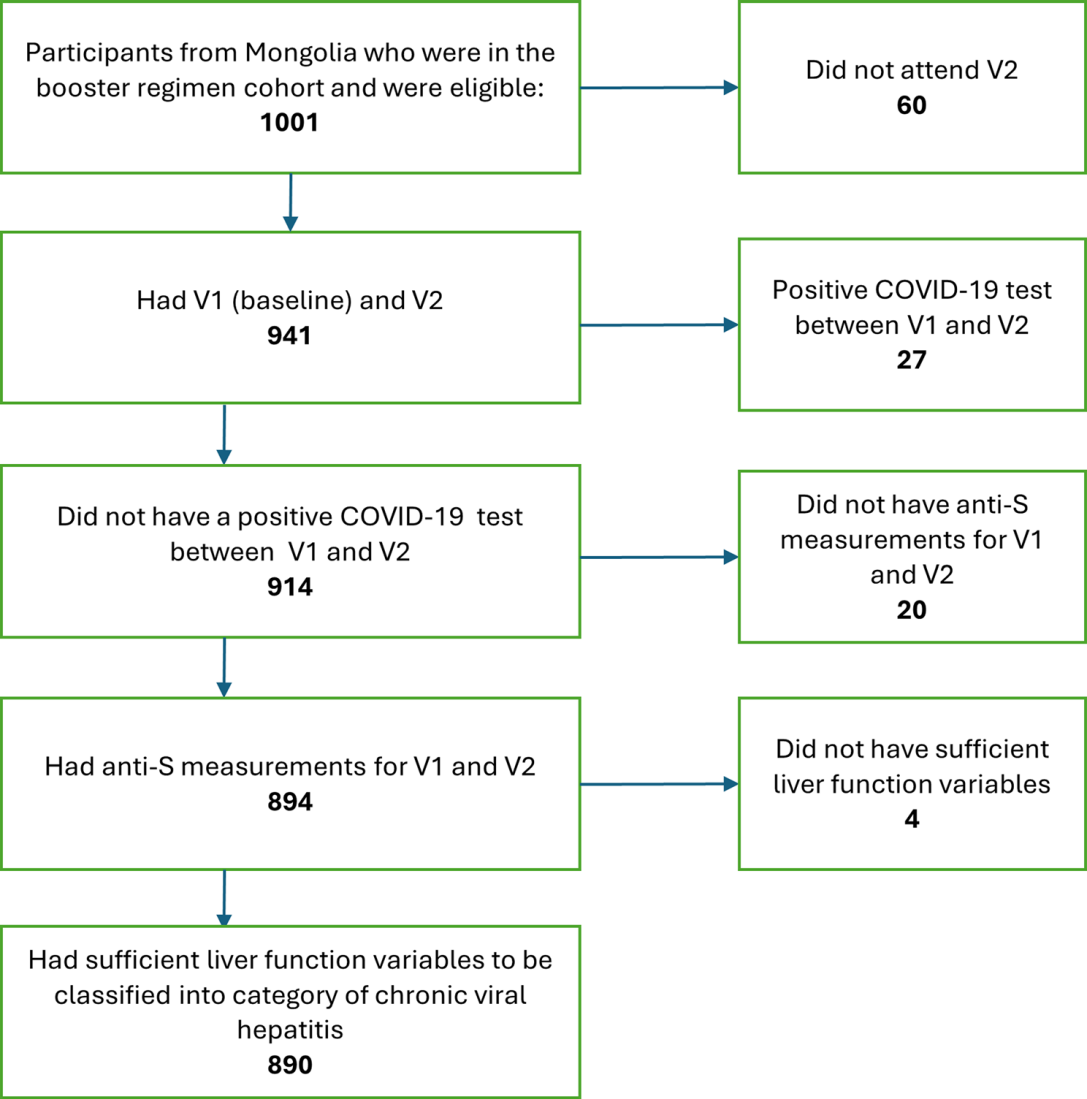
Consort diagram explaining how many participants were eligible and how many ended up included in the analysis dataset

A total of 1001 participants were enrolled into the study in Mongolia. In this analysis we excluded participants that did not attend visit 2 (*n* = 60), had a positive COVID-19 test between visit 1 and visit 2 (*n* = 27), did not have anti-S antibody level measurements at visit 2 (*n* = 22), or did not have liver function laboratory data collected (*n* = 4). After these exclusions, there were 890 persons included in this analysis (Fig. 2). Table 1 includes the demographic characteristics of the participants. The mean age was 45 years and 60% were women. The mean time from vaccine to visit 2 was 95 days for participants with chronic hepatitis and 107 days for participants without chronic viral hepatitis. Upon enrollment, 846 (95%) of participants received Pfizer/BioNTech, 40 (4.5%) received Sinopharm, and 4 (0.4%) received AstraZeneca as a booster vaccine. In the two vaccine doses received prior to our study, 551 participants received at least one dose of Sinopharm and 553 received at least one dose of Pfizer. Other vaccines received in the two doses prior to the study included Gam-COVID-Vac (Sputnik), Bharat Biotech and AstraZeneca. 21% of participants were obese (BMI > = 30). Chronic viral hepatitis infection was present in 169 participants (19%). Of those with chronic hepatitis, 24 (14%) had chronic HCV (alone), 78 (46%) had chronic HBV (alone), and 67 (40%) had chronic HBV/HDV coinfection (Supp. Table 1). No person had chronic infection with both HBV and HCV.

A total of 18 participants had cirrhosis, defined as a Fibroscan score of F4. Eight of 623 (1.3%) participants without chronic viral hepatitis had cirrhosis, and 10 of 152 (6.6%) of participants with chronic viral hepatitis had cirrhosis. Aspartate aminotransferase (AST), alanine aminotransferase (ALT), bilirubin, and albumin were measured only in participants with chronic viral hepatitis. Of the 169 participants with chronic viral hepatitis, 13 (8%) had elevated AST, 32 (19%) had elevated ALT, 21 (12%) had elevated bilirubin, and 3 (1.8%) had decreased albumin, (Supp. Table 2).

**Table 1:** Baseline demographics, overall and by chronic hepatitis status. Number (%) are given for categorical variables.The mean and standard deviation are given for continuous variables

Characteristic	Overall *N* = 890	No chronic viral hepatitis *N* = 721	Chronic viral hepatitis *N* = 169
Age, mean (sd)	45 (11)	45 (12)	46 (10)
Age Category			
18–23	8 (0.9%)	8 (1.1%)	0 (0%)
24–40	326 (37%)	271 (38%)	55 (33%)
41–59	459 (52%)	362 (50%)	97 (57%)
> 59	97 (11%)	80 (11%)	17 (10%)
Booster Vaccine Type			
AstraZeneca/Oxford	4 (0.4%)	4 (0.6%)	0 (0%)
Pfizer/BioNTech	846 (95%)	684 (95%)	162 (96%)
Sinopharm	40 (4.5%)	33 (4.6%)	7 (4.1%)
Sex			
F	533 (60%)	443 (61%)	90 (53%)
M	357 (40%)	278 (39%)	79 (47%)
Body Mass Index (kg/m^2^)			
< 18.5	7 (0.8%)	7 (1.0%)	0 (0%)
>= 18.5 and < 25	343 (39%)	272 (38%)	71 (42%)
>= 25 and < 30	357 (40%)	289 (40%)	68 (40%)
>= 30	183 (21%)	153 (21%)	30 (18%)
Number of prior vaccines			
1	2 (0.2%)	2 (0.3%)	0 (0%)
2	180 (20%)	144 (20%)	36 (21%)
3	708 (80%)	575 (80%)	133 (79%)
Number of participants who received at least one of the following vaccines in the 2 doses prior to study (*some participants received more than 1 type of vaccine)			
AstraZeneca/Oxford	296	253	43
Bharat Biotech	1	1	0
Gam-COVID-Vac (Sputnik)	19	16	3
Pfizer/BioNTech	553	443	110
Sinopharm	551	436	115
Log10 Anti-S titer at visit 1	4.86 (0.60)	4.84 (0.59)	4.92 (0.65)
Time between baseline and visit 2, categories			
< 30 days	1 (0.1%)	1 (0.1%)	0 (0%)
30–59 days	49 (5.5%)	28 (3.9%)	21 (12%)
60–89 days	319 (36%)	246 (34%)	73 (43%)
90–119 days	252 (28%)	217 (30%)	35 (21%)
120–149 days	153 (17%)	129 (18%)	24 (14%)
> 150 days	116 (13%)	100 (14%)	16 (9.5%)
Liver stiffness (F score)			
Not measured	115 (13%)	98 (14%)	17 (10%)
F0-F1	695 (78%)	575 (80%)	120 (71%)
F2	45 (5.1%)	30 (4.2%)	15 (8.9%)
F3	17 (1.9%)	10 (1.4%)	7 (4.1%)
F4 (cirrhosis)	18 (2.0%)	8 (1.1%)	10 (5.9%)
Cirrhosis			
Not measured	115 (13%)	98 (14%)	17 (10%)
Cirrhosis (F4 score)	18 (2.0%)	8 (1.1%)	10 (5.9%)
No cirrhosis (F0-F3 score)	757 (85%)	615 (85%)	142 (84%)

**Table 2:** Estimated differences of log10 anti-S levels at visit 2 between groups (95% confidence interval around difference)

Comparisons: Liver diseased vs. “healthy”	Simple model	Adjusted model
Chronic viral hepatitis (any) vs. none	0.06 (-0.02, 0.14), *N* = 890, *p* = 0.146	0.04 (-0.03, 0.11), *N* = 889, *p* = 0.252
Chronic HCV vs. none *	**0.81 (0.31**,** 1.32)**, *N* = **745**, *p* = **0.002**	**0.37 (0.13**,** 0.60)**, *N* = **745**, *p* = **0.002**
Chronic HBV and HDV coinfection vs. no chronic viral hepatitis	0.02 (-0.10, 0.14), *N* = 788, *p* = 0.691	-0.01 (-0.11, 0.09), *N* = 787, *p* = 0.889
Chronic HBV vs. no chronic viral hepatitis	0.02 (-0.09, 0.13), *N* = 799, *p* = 0.716	0.04 (-0.06, 0.13), *N* = 798, *p* = 0.463
Fibroscan F4 vs. F0-F3	0.19 (-0.03, 0.41), *N* = 773, *p* = 0.094	0.10 (-0.09, 0.28), *N* = 772, *p* = 0.319
Subgroup analyses of those with Chronic viral hepatitis		
Elevated AST vs. not	0.46 (0.16, 0.76), *N* = 169, *p* = 0.003	0.30 (0.04, 0.55), *N* = 169, *p* = 0.022
Elevated ALT vs. not	-0.04 (-0.25, 0.17), *N* = 169, *p* = 0.724	-0.06 (-0.24, 0.11), *N* = 169, *p* = 0.487
Elevated bilirubin vs. not	0.17 (-0.07, 0.42), *N* = 169, *p* = 0.162	0.10 (-0.12, 0.31), *N* = 169, *p* = 0.382
Decreased albumin vs. not	0.28 (-0.34, 0.90), *N* = 169, *p* = 0.371	0.22 (-0.30, 0.73), *N* = 169, *p* = 0.415
**Other subgroup analyses**		
Chronic HBV and HDV coinfection vs. chronic HBV alone	0.01 (-0.16, 0.19), *N* = 145, *p* = 0.886	-0.03 (-0.19, 0.13), *N* = 145, *p* = 0.722
Cirrhosis with chronic hepatitis vs. cirrhosis without chronic hep	0.45 (0.07, 0.83), *N* = 18, *p* = 0.024	0.19 (-0.46, 0.83), *N* = 18, *p* = 0.523

Models with significant interactions are noted by ^*^. For the models with a significant interaction, we show the estimate and 95% CI’s around the difference in groups at time 60 (note this is not a global test of the difference between groups). In the table N represents number included in the analysis, “Simple model” adjusts for time between visit 1 and visit 2. “Adjusted model” adjusts for time between visit 1 and visit 2, anti-S titer at visit 1, anti-N titer at visit 1, number of prior vaccines (before start of study), vaccine type at visit 1, sex, BMI, age, and time from last pre-study vaccine to visit 1

**Fig. 3:**
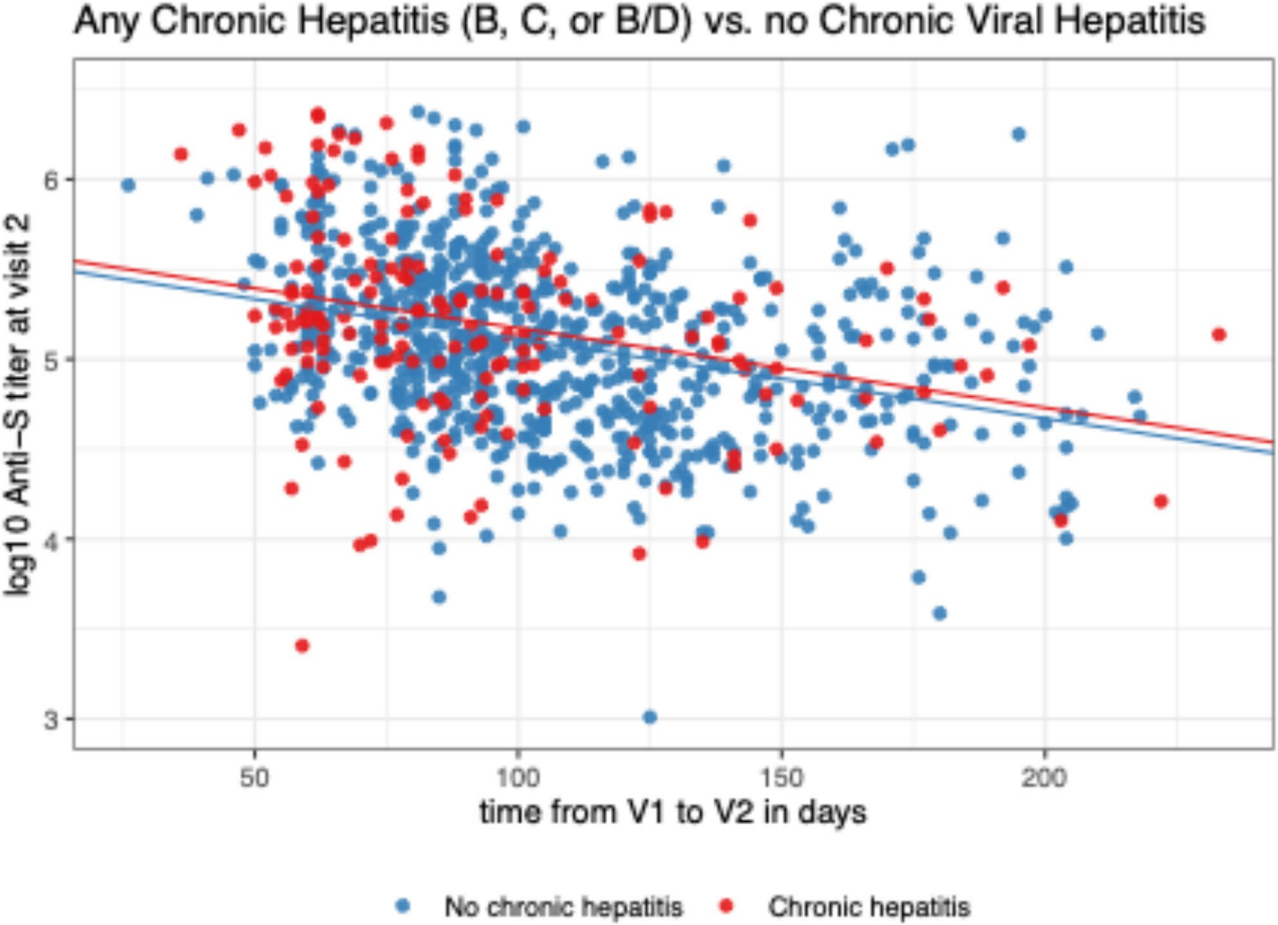
Comparison of anti-S antibody levels between participants with or without chronic viral hepatitis

There was no significant difference in the anti-S antibody response of participants who had any chronic viral hepatitis versus those without chronic viral hepatitis (*p* = 0.252, Fig. 3; Table 2). The only model where there was a significant interaction with time was the model comparing participants with chronic HCV to participants without chronic viral hepatitis. This result was similar in the simple model and the model adjusting for other baseline covariates.

**Fig. 4:**
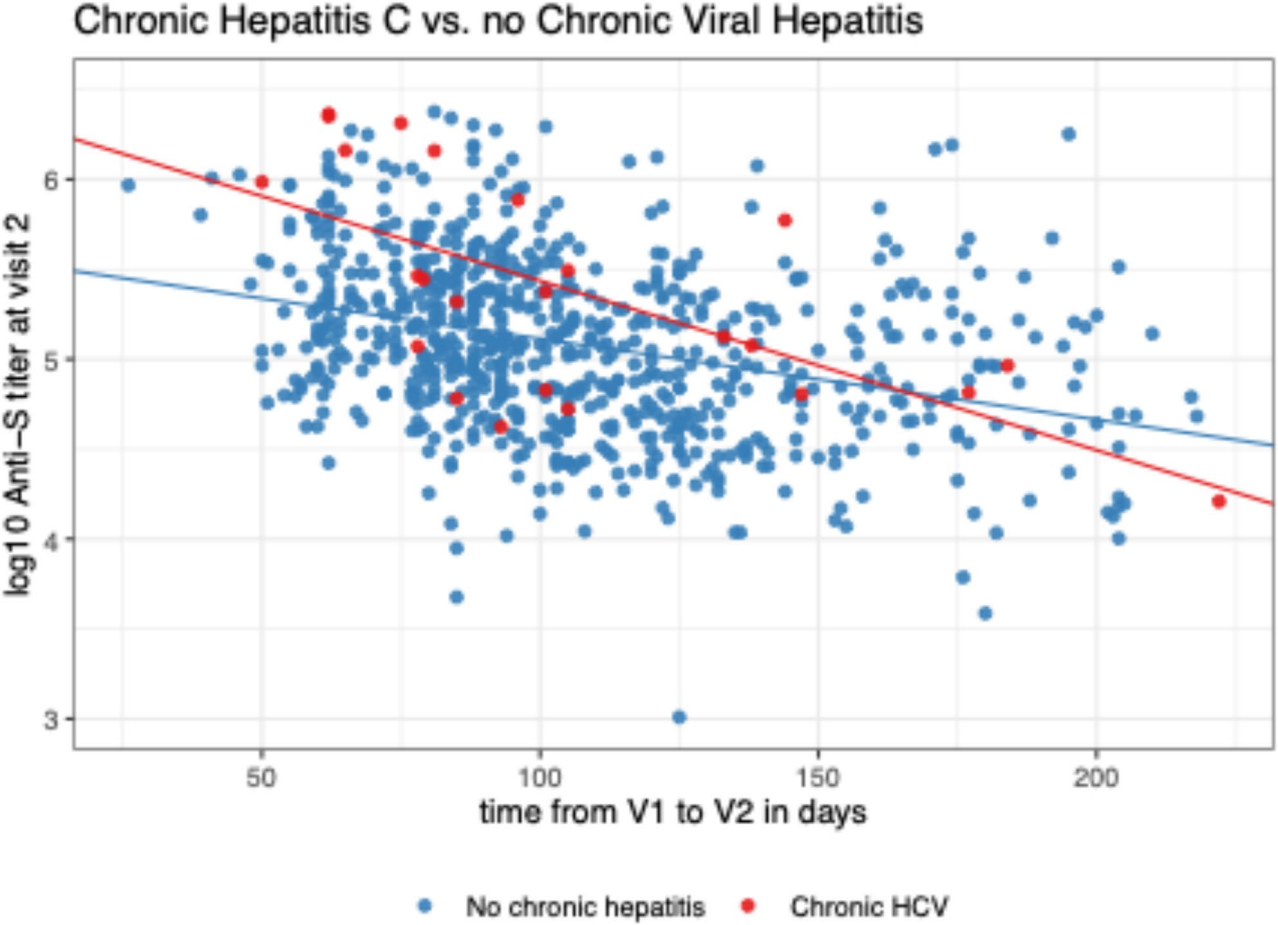
Comparison of anti-S levels in participants with chronic hepatitis C or without chronic viral hepatitis

Because there was a significant time interaction for the model comparing chronic HCV and the control group, we tested for differences in anti-S values at specific points in time. Since Visit 2 was to occur at 60 days (+/- 30 days) following vaccine, we tested for differences between groups at day 60. At day 60, the chronic HCV group had higher anti-S values compared to the controls without chronic viral hepatitis (0.37, CI 0.13–0.60). The interaction indicates the anti-S decrease over time was different in the two groups with the value waning more slowly in those without chronic viral hepatitis compared to the group with chronic HCV (Fig. 4; Table 2). The slope for the control group in the adjusted model was − 0.0036 (-0.0044, -0.0028). The slope for the HCV group in the adjusted model was − 0.0076 (-0.011, -0.0039).

Similar to the overall analysis, no difference was detected in the antibody response of participants with chronic HBV vs. those without chronic viral hepatitis (*p* = 0.463, Table 2) or in the antibody response between participants who had chronic HBV/HDV coinfection compared to those with chronic HBV infection alone (*p* = 0.722, Table 2). Among participants with any type of chronic viral hepatitis, there was no difference in the antibody response between those with or without elevated AST, elevated ALT, elevated bilirubin, or decreased albumin (Table 2). Results were similar in the models that adjusted for baseline covariates. Abnormal AST, ALT, bilirubin or albumin were not significantly associated with anti-S in chronic viral hepatitis participants. Cirrhosis in the chronic viral hepatitis subgroup was significantly related to anti-S values in the simple model, however, this difference disappeared after adjustment for relevant covariates.

## Discussion

Here, we provide an analysis of the anti-S antibody response to COVID-19 booster vaccination in a cohort of Mongolian participants with and without chronic viral hepatitis. As a whole, participants with and without chronic viral hepatitis produced a robust antibody response to the COVID-19 booster vaccine, adding to evidence that COVID-19 vaccines are immunogenic in participants with chronic viral hepatitis. We found no difference in the anti-S response between participants with and without cirrhosis (although only 18 participants had cirrhosis, so the sample size was small) and with and without chronic HBV (48 participants had chronic HBV infection alone).

One difference we did detect was between participants with chronic HCV and participants without chronic viral hepatitis. Those with chronic HCV had a robust antibody response to the COVID-19 booster, but their antibodies waned slightly faster, raising the issue of potential benefit from more frequent vaccinations. This finding needs to be viewed as exploratory given the number of comparisons that were made. This observation may be due to the immune impact of chronic HCV infection that can impair B and T cell responses to immune stimuli [28].

Limitations of our study include the assessment solely for chronic viral hepatitis and not for other causes of liver disease. As the population in this study was recruited in part from a center that treated patients with known liver disease, it was not representative of the general population. Some persons classified as having chronic viral hepatitis could have had acute infection and this was not determined as serial testing for markers of viral hepatitis was not performed. Biochemical liver-related tests were only performed for those with either HBsAg or anti-HCV so that it was not possible to examine the association of abnormal tests in those with and without chronic viral hepatitis or ascertain other etiologies of liver dysfunction. In contrast, all participants had a Fibroscan performed. As only 8 persons without chronic viral hepatitis had a score of F4, the prevalence of advanced liver disease in the group without chronic viral hepatitis appears to be low and cirrhosis, as defined by a score of F4, was seen in only 2%, making assessment of antibody response in this group difficult.

Earlier studies have demonstrated that persons with HBV/HDV coinfection may have more rapidly progressive and severe liver disease compared to those with other forms of chronic viral hepatitis [25]. Because of the relatively high prevalence of HBV/HDV coinfection in Mongolia and in this cohort, a unique feature of this study is the inclusion of those with HBV/HDV coinfection in the cohort. In this analysis, we saw no difference in the anti-S response in this group, something that has not been previously reported (Supp. Table 1). Typically, a proportion of people with chronic hepatitis B or C may develop cirrhosis over time, but the majority do not. In this study population, a relatively low proportion (6.6%) of people with chronic viral hepatitis have cirrhosis.

Other studies found evidence that chronic hepatitis infections and liver cirrhosis negatively impact the immune response to the COVID-19 vaccines. A 2022 multi-center study in China showed that in response to inactivated SARS-CoV-2 vaccines, participants with chronic liver disease produced lower antibody titers and had a lower rate of seroconversion than healthy controls (~ 77% vs. 90%) [1]. In another cohort of Chinese participants with chronic HBV who received BBIBP-CorV and CoronaVac COVID-19 vaccines, those with cirrhosis had lower antibody titers, a slower rate of seropositivity conversion, and a more rapid decline of antibodies over time, than non-cirrhotic patients [4]. Differences between these studies and our study include the fact that most of the participants in our cohort received mRNA vaccines rather than inactivated SARS-CoV-2 vaccines. In addition, our cohort was relatively healthy, with only 18 (2%) participants with cirrhosis, whereas the cohort in Ai et al., [1] had 153 (26%) participants with cirrhosis and in the study by Cao et al., [4], 227 (35%) participants had cirrhosis. Thus, our study was underpowered to detect potentially relevant differences in antibody levels between participants with and without cirrhosis.

Consistent with our findings, several other studies in China [16, 33, 34], as well as studies in Germany [26], Greece [3], and a meta-analysis of 28 observational studies of patients with chronic liver disease or liver transplant [7], concluded that COVID-19 vaccines were safe and immunogenic in patients with chronic liver disease. These studies found that participants with chronic liver disease, chronic HBV or cirrhosis produced robust antibody responses to the vaccine, mostly indistinguishable from healthy controls.

## Conclusions

Our findings strengthen the evidence that the COVID-19 vaccines are immunogenic in people with chronic viral hepatitis, including in those with HBV/HDV coinfection. Future work could extend the follow-up period and consider antibody measurements beyond 90 days.

## Electronic supplementary material

Below is the link to the electronic supplementary material.

Supplementary Material 1

## Data Availability

The datasets used and analyzed during the current study are available from the corresponding author on reasonable request.
