# Supplementary material for: COVID-19 vaccine immunogenicity in Mongolian adults with and without chronic hepatitis
Source: BMC Infect Dis. 2026 Mar 24;26:866. doi: 10.1186/s12879-026-13095-y (PMC13134319; doi:10.1186/s12879-026-13095-y)
Supplement: Supplementary file 1 — Supplementary Material 1 [file 12879_2026_13095_MOESM1_ESM.docx]

**Supplementary material**

Supp. Table 1: Classification by presence and type of chronic viral hepatitis in the cohort. Number of participants (%) are shown.

| **Characteristic** | **Overall** N = 890 | **No chronic viral hepatitis** N = 721 | **Chronic viral hepatitis** N = 169 |
| --- | --- | --- | --- |
| Chronic HCV |  |  |  |
| Chronic HCV infection | 24 (2.7%) | 0 (0%) | 24 (14%) |
| Cleared HCV infection | 89 (10%) | 83 (12%) | 6 (3.6%) |
| No chronic HCV infection | 777 (87%) | 638 (88%) | 139 (82%) |
| Chronic HBV |  |  |  |
| Chronic HBV infection alone | 48 (5.4%) | 0 (0%) | 48 (28%) |
| Chronic HBV/HDV coinfection | 67 (7.5%) | 0 (0%) | 67 (40%) |
| Chronic HBV/resolved HDV | 30 (3.4%) | 0 (0%) | 30 (18%) |
| No chronic HBV infection | 745 (84%) | 721 (100%) | 24 (14%) |
| Consolidated groups |  |  |  |
| No chronic hepatitis | 721 (81%) | 721 (100%) | 0 (0%) |
| Chronic HBV | 78 (8.8%) | 0 (0%) | 78 (46%) |
| Chronic HCV | 24 (2.7%) | 0 (0%) | 24 (14%) |
| Chronic HBV/HDV | 67 (7.5%) | 0 (0%) | 67 (40%) |

Supp. Table 2: Biomarkers measured on participants with chronic viral hepatitis. The table shows numbers of participants (%) outside the normal range and in the normal range.

| **Characteristic** | **Overall** | **abnormal** | **normal** |
| --- | --- | --- | --- |
| ALT | 169 | 32 (19%) | 137 (81%) |
| AST | 169 | 13 (8%) | 156 (92%) |
| bilirubin | 169 | 21 (12%) | 148 (88%) |
| albumin | 169 | 3 (2%) | 166 (98%) |
